# Handling and novel object recognition modulate fear response and endocannabinoid signaling in *nucleus basalis magnocellularis*

**DOI:** 10.1111/ejn.15642

**Published:** 2022-03-17

**Authors:** Iker Bengoetxea de Tena, Marta Moreno‐Rodríguez, Alberto Llorente‐Ovejero, Sergio Monge‐Benito, Jonatan Martínez‐Gardeazabal, Iban Onandia‐Hinchado, Ivan Manuel, Lydia Giménez‐Llort, Rafael Rodríguez‐Puertas

**Affiliations:** ^1^ Department of Pharmacology, Faculty of Medicine and Nursing University of the Basque Country (UPV/EHU) Leioa Spain; ^2^ Department of Audiovisual Communication and Advertising, Faculty of Social Sciences and Communication University of the Basque Country (UPV/EHU) Leioa Spain; ^3^ Department of Clinical and Health Psychology and Research Methodology, Faculty of Psychology University of the Basque Country (UPV/EHU) Leioa Spain; ^4^ Department of Neurodegenerative Diseases BioCruces Bizkaia Health Research Institute Barakaldo Spain; ^5^ Department of Psychiatry and Forensic Medicine, School of Medicine & Institute of Neuroscience Autonomous University of Barcelona (UAB) Barcelona Spain

**Keywords:** aversive memory, CB_1_ mediated‐signaling, cholinergic receptors, functional autoradiography, passive avoidance

## Abstract

Storage of aversive memories is of utmost importance for survival, allowing animals to avoid upcoming similar stimuli. However, without reinforcement, the learned avoidance response gradually decreases over time. Although the molecular mechanisms controlling this extinction process are not well known, there is evidence that the endocannabinoid system plays a key role through CB_1_ receptor‐mediated modulation of cholinergic signaling. In this study, we measured fear extinction throughout 7 months using naïve rats, assessed in passive avoidance (PA) test in a non‐reinforced manner. Then, we evaluated the effect of gentle handling and non‐aversive novel object recognition test (NORT) on the extinction and expression of fear memories by measuring passive avoidance responses. Neurochemical correlates were analyzed by functional autoradiography for cannabinoid, cholinergic, and dopaminergic receptors. Despite results showing a gradual decrease of passive avoidance response, it did not fully disappear even after 7 months, indicating the robustness of this process. Meanwhile, in rats that received gentle handling or performed NORT after receiving the PA aversive stimulus, extinction occurred within a week. In contrast, gentle handling performed before receiving the aversive stimulus exacerbated fear expression and triggered escape response in PA. The neurochemical analysis showed increased cannabinoid and cholinergic activity in the *nucleus basalis magnocellularis* (NBM) in rats that had performed only PA, as opposed to rats that received gentle handling before PA. Additionally, a correlation between CB_1_ mediated‐signaling in the NBM and freezing in PA was found, suggesting that the endocannabinoid system might be responsible for modulating fear response induced by aversive memories.

## Graphical abstract

Gentle handling performed to naïve rats before receiving an aversive stimulus exacerbates fear in a subsequent PA exposure, whereas NORT previous to an aversive stimulus reduces passive avoidance response. Freezing, the main indicator of fear response in PA, is reduced in groups that performed handling or NORT before PA, and it correlates with CB_1_ mediated‐signaling in the NBM. The endocannabinoid system might be responsible for these modulations of fear responses.

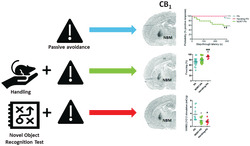

**Abbreviations**
BLA: basolateral amygdala
BSA: bovine serum albumin
DTT: DL‐dithiothreitol
eCB: endocannabinoid
GDP: guanosine 5′‐diphosphate
GTPγS: guanosine 5′‐O‐3‐thiotriphosphate
mPFC: medial prefrontal cortex
NBM: *nucleus basalismagnocellularis*
NORT: novel object recognition test
PA: passive avoidance
VTA: ventral tegmental area

## 1 INTRODUCTION

Aversive stimuli induce changes in behavior that can lead to fight‐or‐flight responses. The behavioral responses after an aversive stimulus vary across animal species and depend on the context and factors such as the possibility to escape from the threat (Stahl, 2015). For the study of aversive stimuli, conditioned fear paradigms are usually used, and a decline in fear response is often observed following non‐reinforced exposure to the feared conditioned stimulus, a phenomenon known as “fear extinction” (Myers et al., 2006). In such tests, freezing behavior is often measured as an indicator of fear. Besides that, active and passive avoidance (PA) tests are also useful for the assessment of fear response in rodents. In active avoidance tests, rodents learn to avoid an aversive stimulus, in the form of a mild foot shock, by initiating a specific locomotor response (Wadenberg, 2014). In PA, rodents learn to avoid the aversive stimulus by adopting a freezing behavior (passive avoidance response) (Bourin & Hascoët, 2003). This test is widely used to evaluate the effect of new drugs for the treatment of cognitive and memory deficits, such as those associated with cholinergic signaling, as well as to characterize new genetic and surgical animal models of dementia and cognitive impairment in rodents (Ogren et al., 2015).

Certain non‐pharmacological procedures, such as handling, can modulate the acquisition, expression, and extinction of aversive memories. Results from elevated plus maze test, which is used to test the levels of anxiety of rodents, indicate that rats that have been gently handled before the test have decreased anxiety levels, which results in improved learning and memory (Boix et al., 1988; Costa et al., 2012; Fernández‐Teruel et al., 1991). The same effect of handling has been observed in neonatal rodents, in fear‐motivated tests such as the afore‐mentioned active avoidance, among others (Muntsant et al., 2019; Río‐Álamos et al., 2017; Siviy, 2018).

Similarly, environmental enrichment produces profound changes in the expression of fear and anxiety in rodents (Fernández‐Teruel et al., 2002). Rats hosted in environmentally enriched cages have reduced levels of anxiety in the open field test (Hines et al., 2012), and these environments also impact neurotransmission in the medial prefrontal cortex (mPFC), resulting in an accelerated extinction of fear memories (Marek et al., 2018).

Although the potential influence of certain behavioral procedures on the performance of subsequent tests has been reported before (Blokland et al., 2012; Gururajan et al., 2010; McIlwain et al., 2001; von Kortzfleisch et al., 2019), the use of behavioral batteries that include several consecutive tests is a usual approach for the evaluation of fear, anxiety, cognition, and learning in rodents (Blanchard et al., 2003; Wolf et al., 2016; You et al., 2019). Many of the tests used for these purposes, such as elevated plus‐maze test, open field test, or Morris water maze, have an anxiogenic component, whereas others, such as novel object recognition test (NORT), are stimulating in a way similar to environmental enrichment, possibly altering the basal behavior of the rodents. These observations raise the question of the suitability of using the same group of animals to perform several continuous behavioral tests.

The response of rodents to fear and its modulation by different procedures, leading to active or passive coping strategies, is mediated by specific brain circuits involving several areas (Keay & Bandler, 2001). Projections from the amygdala and hippocampus to the ventral tegmental area (VTA) play a key role in the acquisition and expression of fear, but other areas, such as the nucleus accumbens and the mPFC, also modulate this process (Albrechet‐Souza et al., 2013). Local dopamine release in these areas is involved with the expression of passive coping strategies (Guarraci et al., 1999), and also in the consolidation and long‐term storage of the aversive memories that trigger such responses (Kramar et al., 2020). In the extinction of fear memories, the mPFC plays a key role, as pyramidal neurons in layers V‐VI of the mPFC project to pyramidal cells in layers V‐VI of the infralimbic cortex, contributing to the enhancement of this process (Marek et al., 2018). Besides the involvement of dopaminergic activity, this fear‐control pathway is also influenced by endogenous cholinergic inputs to the basolateral amygdala (BLA) and the hippocampus from the *nucleus basalis magnocellularis* (NBM) (Knox, 2016). Endocannabinoid (eCB) modulation of this process is also required (Riebe et al., 2012). In fact, a crosstalk between cholinergic and endocannabinoid neurotransmission plays a role in the modulation of fear extinction. CB_1_‐deficient mice show strongly impaired fear extinction in auditory fear‐conditioning tests, in the short‐ and the long‐term, but memory acquisition and consolidation remain unaffected (Marsicano et al., 2002). Moreover, pharmacological modulation of eCB signaling leads to behavioral changes in the expression of fear. For instance, sub‐chronic CB_1_ stimulation in limbic areas can inhibit the increase of fear inherent to adult 3xTg‐AD mice, a transgenic model of familial Alzheimer's disease (Llorente‐Ovejero et al., 2018).

When performing novelty‐seeking memory tests, such as NORT, followed by aversive memory tests with an anxiogenic component, such as PA, using naïve Sprague–Dawley male rats, we observed unexpected behaviors. For instance, control rats crossed to the black compartment on retention phase, contrary to the expected passive avoidance response (unpublished results). To further study this phenomenon and the possible effect of novelty‐seeking and factors such as animal handling on passive avoidance response, we evaluated fear extinction and fear expression in PA test through (1) temporal patterns (7 months, in a non‐reinforced manner), (2) exposure to environmental interactions involving tactile‐proprioceptive stimulation (handling) and novelty‐seeking (NORT) after receiving an aversive stimulus (fear extinction), (3) the spontaneous elicitation of learning and memory related to handling and novelty‐seeking (NORT) before receiving the aversive stimulus (fear expression). Finally, we aimed to study the neurochemical correlates of these behaviors. For that, we used functional receptor autoradiography to analyze the state of the dopaminergic, cholinergic, and endocannabinoid systems in brain areas involved in the regulation of fear extinction and expression, including BLA, VTA, mPFC, and NBM.

## 2 MATERIALS AND METHODS

### 2.1 Animals

Ninety nine naïve Sprague–Dawley male rats (200–300 g) were bred and maintained in makrolon (Covestro, Leverkusen, Germany) cages (38.2 × 22.0 cm), in groups of three to four rats per cage, under standard laboratory conditions (food and water ad libitum, 22°C ± 2°C, 12 h light: dark cycle, 65%–70% relative humidity). The experimental protocols regarding the use of laboratory animals were approved by the Local Ethics Committee for animal research of the University of the Basque Country (CEEA M20‐2018‐52 and 54), which is in accordance to EU directive 2010/63/EU for animal experiments.

### 2.2 Chemicals

WIN 55,212–2 was purchased from Tocris Bioscience (Bristol, UK). Carbachol and rotigotine, as well as Kodak Biomax MR β‐radiation‐sensitive films, bovine serum albumin (BSA), DL‐dithiothreitol (DTT), adenosine deaminase, guanosine 5′‐diphosphate (GDP), guanosine 5′‐O‐3‐thiotriphosphate (GTPγS), ketamine and xylazine were purchased from Sigma–Aldrich (St Louis, MO, USA). The [^14^C]‐microscales, used as standards in the autoradiographic experiments, were purchased from ARC (American Radiolabeled Chemicals, St Louis, MO, USA). All the compounds necessary were of the highest commercially available quality.

### 2.3 Behavioral tests

#### 2.3.1 Passive avoidance test (PA)

**FIGURE 1:**
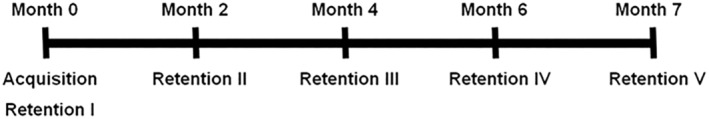
Passive avoidance schedule to evaluate fear extinction after receiving an aversive stimulus, throughout 7 months

**FIGURE 2:**
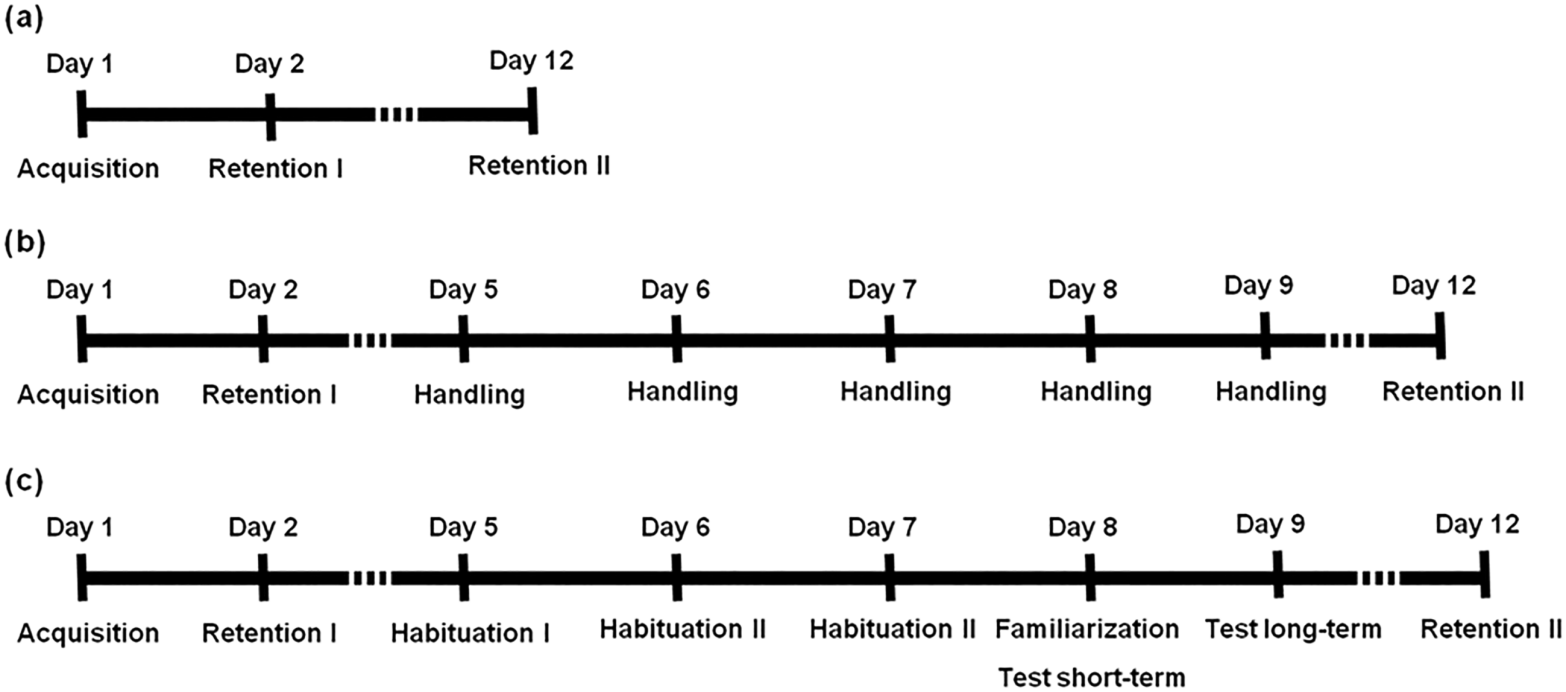
Schedules of the different behavioral batteries administered to the rats to evaluate the effect of handling and non‐aversive memory test NORT on fear extinction. (a) Schedule performed by the control PA‐PA group. (b) Schedule performed by PA‐Handling‐PA group. (c) Schedule performed by PA‐NORT‐PA group

**FIGURE 3:**
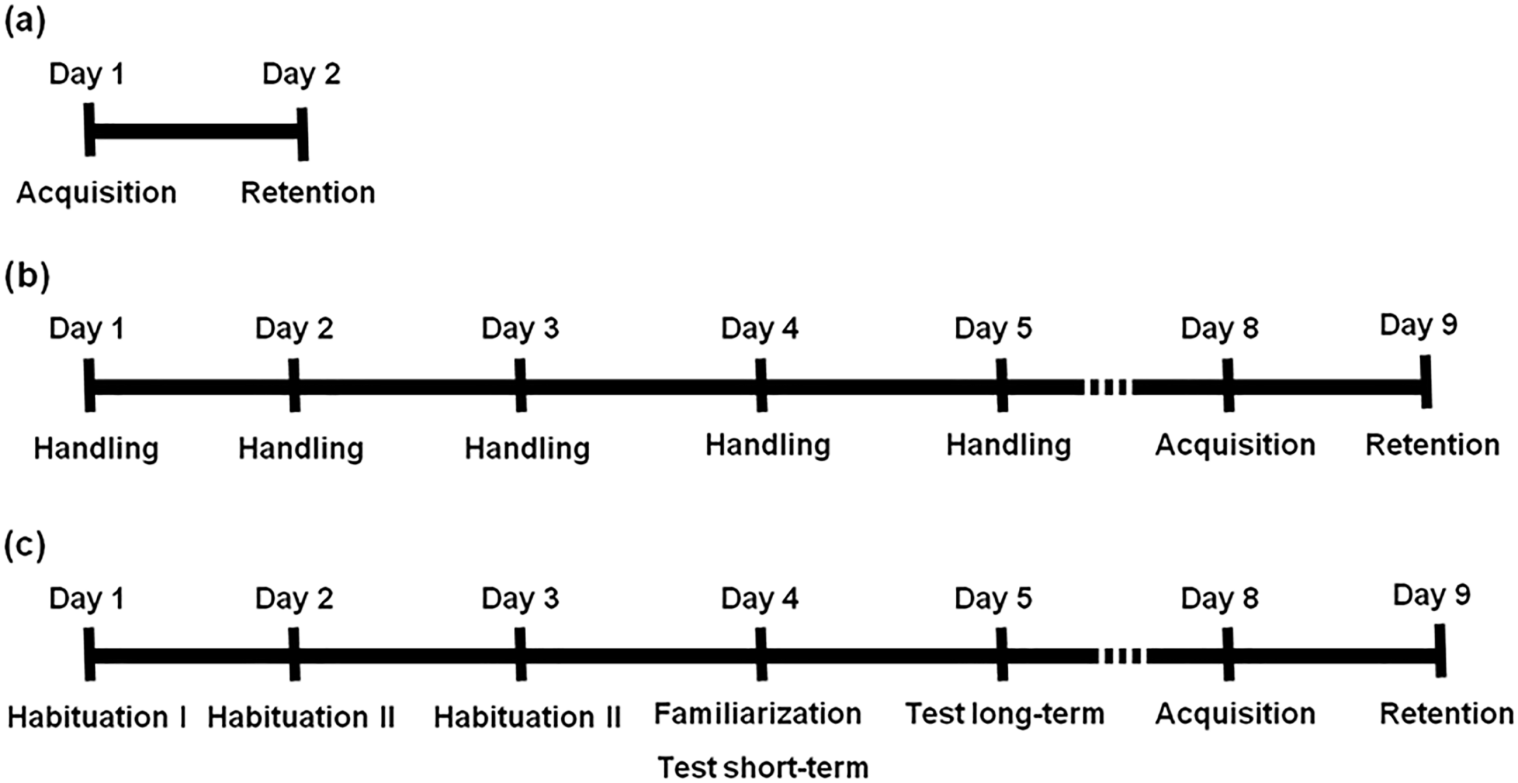
Schedules of the different behavioral batteries administered to the rats to evaluate the effect of handling and non‐aversive memory test NORT on fear expression. (a) Schedule performed by the control PA group. (b) Schedule performed by Handling‐PA group. (c) Schedule performed by NORT‐PA group

To evaluate the decline of fear response and extinction throughout 7 months, a modified protocol of PA was used, with 1 day of acquisition and 1 day of retention 24 h later, and then an additional retention every 2 months (see Figure 1). Additionally, depending on the group, PA was either performed with two retention phases separated by 5 days in which NORT or handling was administered to the rats (see Figure 2) or following a standard two‐day protocol with a single retention phase 24 h after acquisition (see Figure 3). Before each trial, rats were transported to the experimental room for habituation. The shuttle box (PanLab S.L., passive avoidance box LE870/872, Barcelona, Spain) in which the test was performed consisted of two compartments (black, 19.5 × 10.8 × 12 cm and white, 31 × 31 × 24 cm), connected by an automatic gate. A video camera placed above the shuttle box recorded the behavior of the rats. On the first day (acquisition phase), rats were placed in the white compartment. They were allowed to explore the compartment for 30 s before the door connecting the white compartment with the black compartment opened. When the rats innately crossed to the black compartment with all four paws, the door closed and they received a mild foot shock (0.4 mA for 2 s). The rats remained in the black compartment, with the door closed, for 15 s before being carefully returned to their cage. After each rat, the shuttle box was cleaned with 70% ethanol and dried to minimize olfactory cues. Twenty‐four hours later, on the second day (retention phase), the rats were again placed in the white compartment, and the PA response was evaluated. The rats were given 5 min to decide whether to cross to the black compartment or not. Step‐through latency time was measured; higher latencies indicated passive avoidance and were considered a positive response to the test. The total path length of the rats and their speed during the acquisition and probe trial phases were measured using an automated tracking system (SMART, Panlab S.L., Barcelona, Spain), as well as the percentage of freezing time, which was used as an indicator of fear. The number of stretch attendances from the white to the black compartment was measured manually and used as a sign of risk assessment.

#### 2.3.2 Novel Object Recognition Test (NORT)

NORT was performed following a modified internal protocol consisting of 3 days of habituation (habituation phase), 1 day of familiarization (familiarization phase), and two trials, one on the fourth day 3 h after familiarization (short‐term) and one on the fifth day, 24 h after familiarization (long‐term). For the test, a white open‐field arena (90 × 90 × 50 cm) (Panlab S.L., Barcelona, Spain) was used under one lux light condition. Each rat was gently handled individually for 1 min before being subjected to any trial and was transported to the experimental room for habituation. During the habituation phase, the rats were placed in the arena for three consecutive days and were allowed to explore the compartment for 5 min. During the familiarization phase on the fourth day, the rats were presented with two identical objects (object A and object A), built with five to six mega blocks, with a height of about 10 cm. The objects were positioned diagonally, approximately 10 × 10 cm away from their respective walls, and were mirror images of each other. The position of the objects in the arena rotated after each rat, to avoid possible location related bias. The rats were allowed to stay in the arena until they had explored both objects for a total of 25 s during 10 min as a cut‐off time. The rats which failed to reach the 25 s exploration threshold were excluded from the experiment, to ensure a minimum learning of the familiar object. Exploration of the objects was considered when the rats touched the object or faced it with their nose being less than 2 cm away from it. On that same fourth day, 3 h after the familiarization phase, the short‐term testing was carried out as follows: the rats were again placed in the arena and were presented with a familiar object (object A) and a new one (object B). The rats were allowed to explore both objects for 5 min. On the fifth day, 24 h after the familiarization phase, the rats were again placed in the arena for long‐term testing. The new object from the short‐term testing phase (object B) was replaced by a different one (object C). The rats were allowed to explore both objects for 5 min. In this study, NORT is used as an intervention, just like handling, to explore the effect of novelty‐seeking before and after receiving an aversive stimulus. Thus, the parameters from NORT were not measured and consequently are not shown in the present study.

#### 2.3.3 Handling

A group of rats received the standard handling procedure performed as part of NORT protocol during 5 days. Each day, the rats were removed from their home cages and placed in smaller cages. They were transported to the experimental room, where behavioral tests were carried out, under one lux light condition. Each rat was gently handled individually for 1 min, having its neck and back stroked by the experimenter's fingers, and then remained in the experimental room for 10 min. Then, each rat was handled again for 1 min and returned to their home cage.

### 2.4 Behavioral schedules

The design consisted of three experimental sets and procedures where fear response and extinction were evaluated in PA: (1) long‐term non‐reinforced exposure to PA for 7 months to study fear extinction across time, (2) exposure to tactile‐proprioceptive stimulation (handling) and novelty‐seeking (NORT) after receiving an aversive stimulus, to study the modulation of fear extinction by these procedures, (3) exposure to handling and NORT previous to the aversive stimulus, to study the modulation of fear expression by these procedures. Animals from all groups were sacrificed 24 h after finishing their respective experimental procedures.

#### 2.4.1 Temporal pattern of fear extinction

The fear response and extinction were measured (*n* = 10) every 2 months, for 7 months, as a decline of passive avoidance response in the retention phase of the test (see Figure 1). The evaluation was performed every 2 months because we did not know when passive avoidance response would decrease significantly.

#### 2.4.2 Effect of handling and NORT on fear extinction

The effects of animal handling or the performance of NORT on the fear extinction were evaluated using three groups of rats: control PA‐PA group (*n* = 8), PA‐Handling‐PA group (*n* = 11) and PA‐NORT‐PA group (*n* = 12) (see Figure 2).

#### 2.4.3 Effect of handling and NORT on fear response before receiving an aversive stimulus

The effect of animal handling and NORT on the fear response before receiving an aversive stimulus was evaluated by performing a protocol of animal handling or NORT before the acquisition phase of PA. For that, three groups of rats were used: control PA group (*n* = 23), Handling‐PA group (*n* = 12) and NORT‐PA group (*n* = 23) (see Figure 3).

### 2.5 Neurochemical analysis

#### 2.5.1 Functional [^35^S]GTPγS autoradiography

To study the neurochemical basis of the observed behavioral changes in NORT‐PA and Handling‐PA groups, compared to control PA group, G protein‐coupled receptor‐mediated intracellular signaling was analyzed by using functional [^35^S]GTPγS autoradiography. The dopaminergic, cholinergic, and cannabinoid systems were analyzed. Rats were anesthetized (i.p. ketamine/xylazine 90/10 mg/kg), sacrificed, and the brains were carefully removed from the skull before being frozen and stored at −80°C. Later, brains were serially cut in a Microm HM550 cryostat (Thermo, Germany) in 20 μm thick sections, mounted on gelatin‐coated glass slides and stored at −20°C until they were used. For the functional [^35^S]GTPγS autoradiography, the tissue sections were dried for 15 min and then preincubated four times in HEPES‐based buffer (50 mM HEPES, 100 mM NaCl, 3 mM MgCl_2_, and 0.2 mM EGTA, 1% BSA and 0.1% DMSO, pH 7.4) for 15 min each time at 30°C in a water bath. Then, the brain sections were incubated for 2 h in HEPES‐based buffer supplemented with 2 mM GDP, 1 mM DL‐dithiothreitol (DTT) and 0.04 nM [^35^S]GTPγS, at 37°C in a water bath. The basal binding was obtained in the absence of agonists. The agonist‐stimulated binding of dopaminergic, cholinergic and cannabinoid G_i/o_ proteins‐coupled receptors was measured under the same conditions in the presence of the specific agonists rotigotine (10^−5^ M), carbachol (10^−5^ M) and WIN 55,212‐2 (10^−5^ M), respectively. Non‐specific binding was determined in the presence of 10 mM of non‐labeled GTPγS. After the incubation, the sections were washed twice in HEPES buffer (50 mM, pH 7.4) at 4°C for 15 min each time, dipped in distilled water and dried. Finally, sections were exposed to autoradiography films, together with [^14^C] standards, for 48 h at 4°C in hermetically closed cassettes.

#### 2.5.2 Quantitative image analysis of autoradiograms

Films were scanned and quantified by transforming the optical densities into nCi/g of tissue equivalent (nCi/g t.e.) using Fiji software (Fiji, Bethesda, MA, USA). [^14^C] standards were used to calibrate the optical densities with the level of radioactivity labeled to the sections. Experimental data was analyzed by using GraphPad Prism software (GraphPad Software Inc., CA, USA) and Microsoft Excel software (Microsoft, WA, USA). Basal binding was calculated as nCi/g tissue equivalent using the values provided in the [^14^C] microscales. The agonist stimulation of [^35^S]GTPγS binding was expressed by the percentage of stimulation over the basal activity, in the presence of different agonists. It was calculated as: ([^35^S]GTPγS agonist‐stimulated binding) × 100/([^35^S]GTPγS basal binding) − 100.

### 2.6 Statistical analysis

Statistical analysis of data was performed with GraphPad Prism. To analyze step‐through latency of the rats during the retention phase of PA, Kaplan–Meier survival curves were used and the analysis was performed using a log‐rank/Mantel–Cox test. Due to the presence of censored data (cut‐off time for step‐through latency times of 300 s), this type of analysis is the most suitable one and represents an alternative for the interpretation of the behavioral results. Previous studies from our group have used this type of analysis for PA test (Llorente‐Ovejero et al., 2017). It evaluates the probability of the rats to reach the cut‐off time of 300 s (which is considered as a positive response in PA test), with the survival curves depicting the retention time (step‐through latency) of each rat. If the probability to reach the cut‐off time is below 50%, the median latency (the time at which half of the rats enter the black compartment) can be determined. Each Kaplan–Meier's survival curve was individually compared to every other curve. The number of stretch attendances, the percentage of the freezing time, and autoradiograms were analyzed with Kruskal–Wallis test, followed by post‐hoc test Dunn's multiple comparison. Experimental data from the autoradiograms were correlated with the behavioral parameters described above (learning latency, retention latency, number of stretch attendances, and percentage of the freezing time). The correlations were analyzed using a nonparametric two‐tailed Spearman's test. Statistical significance was set at *P* < 0.05.

## 3 RESULTS

### 3.1 Fear extinction in PA is a long‐term process

**FIGURE 4:**
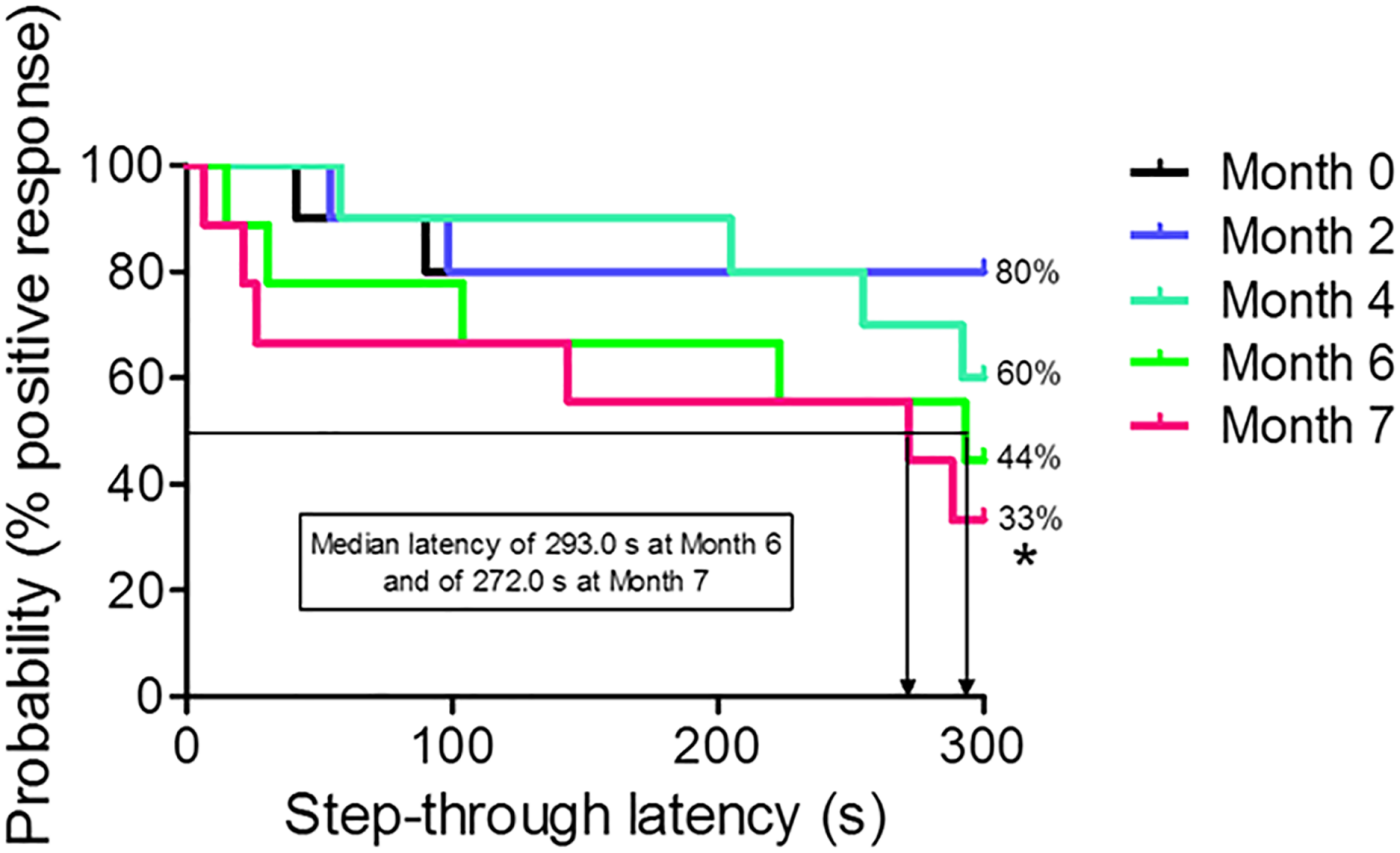
Step‐through latency times of the rats during the retention phase of PA represented as Kaplan–Meier's survival curves (*n* = 10). The rats were removed from the shuttle box after 300 s of cut‐off time. Significant differences were observed between Month 0 and Month 7 (log‐rank/Mantel–Cox test, *P = 0.047 Month 0 vs. Month 7)

Throughout 7 months, the fear extinction process in PA was analyzed in a group of 10 rats. We observed that PA response gradually decreased with time. In the retention trial performed 24 h after the rats received a mild foot shock (0.4 mA for 2 s) in the black compartment, 80% of the rats displayed a positive PA response. Two months later, the percentage of rats that displayed a positive response was still 80%. Four months later, it was 60%. In 6 months, it was 44% and the median latency (i.e., the time that 50% of the rats took to cross the doorway separating both compartments) was 293.0 s. Comparing month 0 versus month 6, *P* value was 0.135, close to statistical significance. We thus performed the next retention 1 month later, instead of two. Finally, after 7 months, 33% of the rats displayed a positive response, and the median latency dropped to 272.0 s. Comparing month 0 versus month 7, *P* value was 0.047. Figure 4 shows that fear extinction, as measured in PA, is a long‐term process, lasting more than 7 months.

### 3.2 Handling and NORT accelerate fear extinction after receiving an aversive stimulus

**FIGURE 5:**
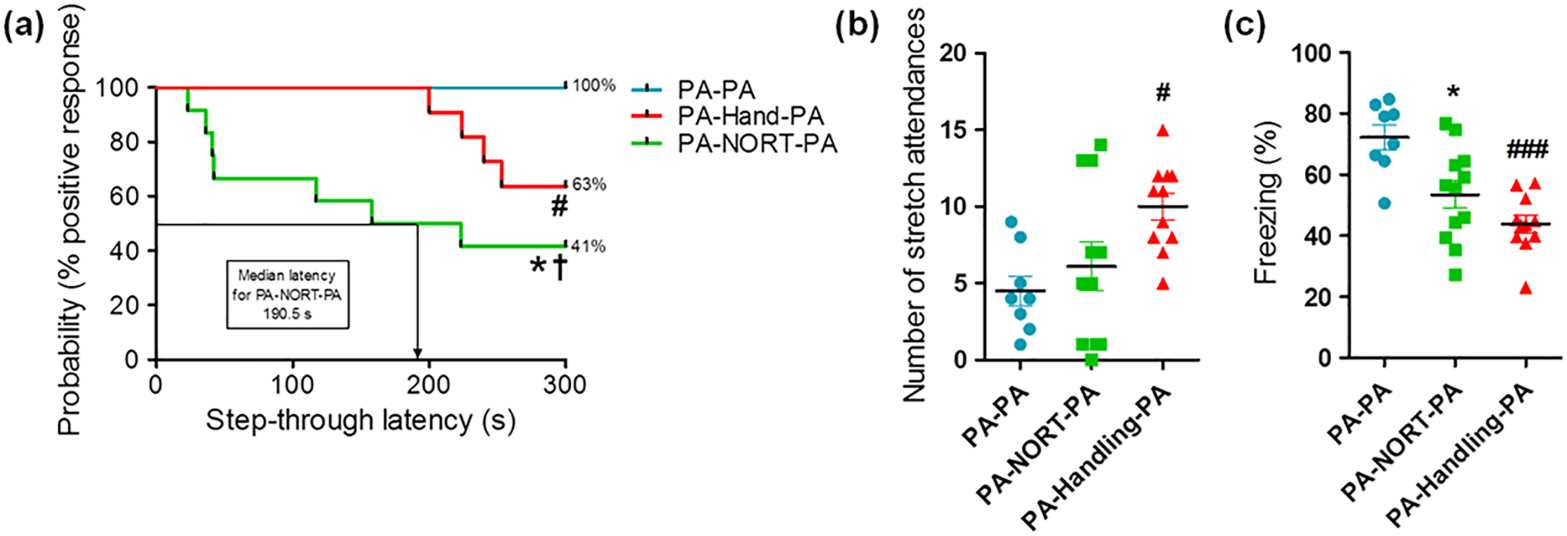
Analysis of the fear response of the rats from PA‐PA (*n* = 8), PA‐Handling‐PA (*n* = 11) and PA‐NORT‐PA (*n* = 12) groups after receiving an aversive stimulus. (a) Step‐through latency times during the retention phase of PA represented as Kaplan–Meier's survival curves. Significant differences were observed between all groups (log‐rank/Mantel–Cox test, *P = 0.011 PA‐PA vs. PA‐NORT‐PA, #*P* = 0.037 PA‐PA vs. PA‐Handling‐PA, †*P* = 0.039 PA‐NORT‐PA vs. PA‐Handling‐PA). (b) Number of stretch attendances during the retention phase of PA. Significant differences were observed between the groups PA‐PA and PA‐Handling‐PA (Kruskal–Wallis test, post‐hoc test Dunn's multiple comparison, H(2) = 7.85, #*P* < 0.05 PA‐PA vs. PA‐Handling‐PA). (c) Percentage of freezing time during the retention phase of PA. Significant differences were observed between the groups PA‐PA and PA‐NORT‐PA and the groups PA‐PA and PA‐Handling‐PA (Kruskal–Wallis test, post‐hoc test Dunn's multiple comparison, H(2) = 13.63, *P < 0.05 PA‐PA vs. PA‐NORT‐PA and ###*P* < 0.001 PA‐PA vs. PA‐Handling‐PA)

Handling of the rats and NORT influenced fear extinction process in PA. When analyzing the behavior of PA‐NORT‐PA and PA‐Handling‐PA groups, significant differences were observed between these groups and the control PA‐PA group in key parameters, such as retention latency (see Figure 5a), number of stretch attendances (see Figure 5b) and percentage of freezing time (see Figure 5c).

The 100% of the rats from PA‐PA group displayed a positive passive avoidance response. In contrast, the 41% of those from PA‐NORT‐PA group displayed a positive passive avoidance response, and the median latency for this group was 190.5 s. Moreover, the percentage of freezing time was also modified with rats from PA‐PA group spending more time freezing than those from PA‐NORT‐PA group (72.25 ± 4.09% vs. 53.49 ± 4.45%, *P* < 0.05).

When comparing PA‐Handling‐PA and PA‐PA groups, statistically significant differences were found in retention latency (see Figure 5a), the number of stretch attendances (see Figure 5b) and the percentage of freezing time (see Figure 5c). In PA‐Handling‐PA group, 63% displayed a positive response during the retention phase. Regarding the number of stretch attendances, rats from PA‐Handling‐PA group showed higher number of stretch attendances during retention phase compared to PA‐PA group (10.00 ± 0.86 vs. 4.50 ± 0.98, *P* < 0.05). Looking at the percentage of freezing time, PA‐Handling‐PA group spent less time freezing than PA‐PA group (43.89 ± 2.89% vs. 72.25 ± 4.09%, *P* < 0.001).

Significant differences were also observed between PA‐NORT‐PA and PA‐Handling‐PA groups in retention latency (see Figure 5a), but not in the number of stretch attendances (see Figure 5b) and the percentage of freezing time (see Figure 5c). 41% of rats from PA‐NORT‐PA group displayed a positive passive avoidance response, whereas 63% of the rats from PA‐Handling‐PA group did so.

### 3.3 Handling of the rats before receiving an aversive stimulus exacerbates fear expression, whereas previous NORT reduces it

**FIGURE 6:**
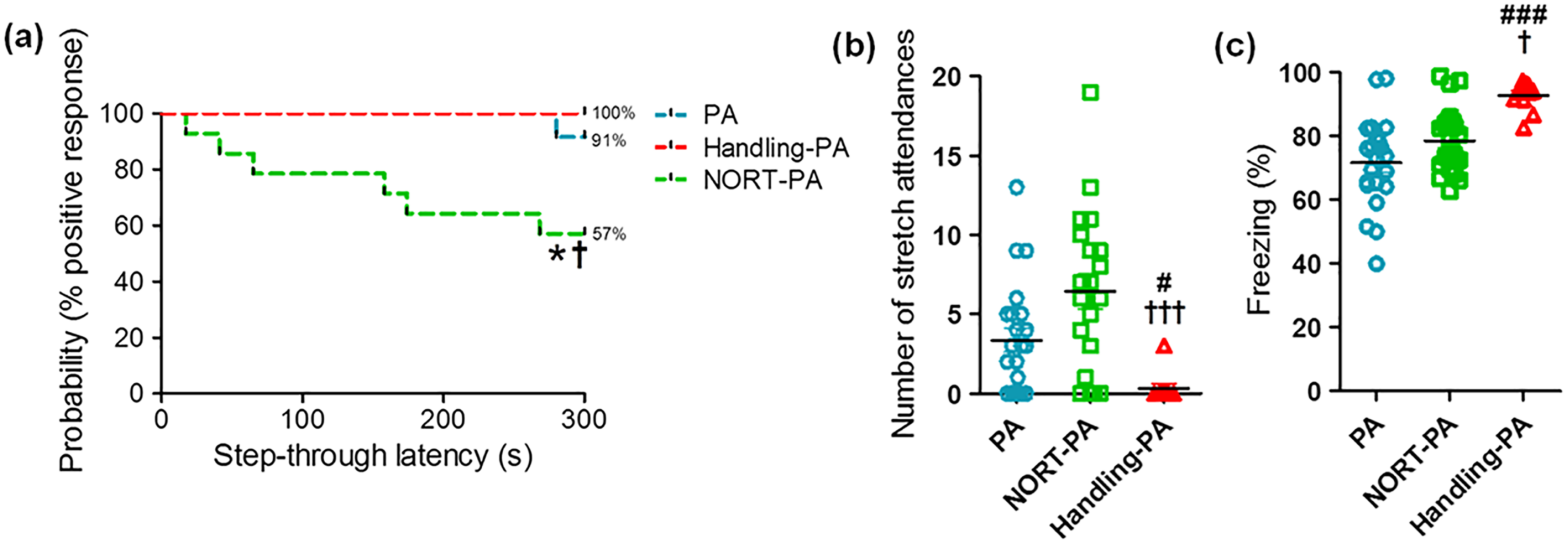
Analysis of the fear response of the rats from PA (*n* = 23), Handling‐PA (*n* = 12), and NORT‐PA (*n* = 23) groups after receiving an aversive stimulus. (a) Step‐through latency times during the retention phase of PA represented as Kaplan–Meier's survival curves. Significant differences were observed between the groups PA and NORT‐PA and the groups NORT‐PA and Handling‐PA (log‐rank/Mantel–Cox test, *P < 0.041 PA vs. NORT‐PA, †*P* < 0.011 NORT‐PA vs. Handling‐PA). (b) Number of stretch attendances during the retention phase of PA. Significant differences were observed between the groups PA and Handling‐PA and the groups NORT‐PA and Handling‐PA (Kruskal–Wallis test, post‐hoc test Dunn's multiple comparison, H(2) = 15.55, #*P* < 0.05 PA vs. Handling‐PA, †††P < 0.001 NORT‐PA vs. Handling‐PA). (c) Percentage of freezing time during the retention phase of PA. Significant differences were observed between the groups PA and Handling‐PA and between the groups NORT‐PA and Handling‐PA (Kruskal–Wallis test, post‐hoc test Dunn's multiple comparison, H(2) = 16.35, ###*P* < 0.001 PA vs. Handling‐PA, †*P* < 0.05 NORT‐PA vs. Handling‐PA)

Handling before receiving an aversive stimulus exacerbates fear expression in the retention phase of PA. When comparing the behavior of the control PA group with Handling‐PA group, significant differences were observed in two key parameters, the number of stretch attendances (see Figure 6b) and the percentage of freezing time (see Figure 6c), but no differences were observed in retention latency (see Figure 6a).

One hundred percent of the rats from Handling‐PA group displayed a positive passive avoidance response, compared to 91% of the rats from PA group (see Figure 6a). Differences were also observed in the number of stretch attendances (see Figure 6b) and the percentage of freezing time (see Figure 6c), with rats from Handling‐PA group performing fewer stretch attendances than those from PA group (0.25 ± 0.25 vs. 3.30 ± 0.72, *P* < 0.001) and presenting a higher percentage of freezing time (93.16 ± 1.41% vs. 72.10 ± 2.90%, *P* < 0.05). Surprisingly, rats from Handling‐PA group also exhibited “escape behavior,” as shown by the fact that up to 83.33% of them tried to jump out of the white compartment at least once, and up to five times, during the retention phase of PA (i.e., 24 h later of the foot shock). This behavior was not observed in any of the other groups.

Conversely, when comparing NORT‐PA and PA groups, significant differences were observed in retention latency (see Figure 6a). The 57% of the rats from NORT‐PA group displayed a positive passive avoidance response in contrast to the 91% of those from PA group. Significant differences were also observed in the number of stretch attendances (see Figure 6b), with rats from NORT‐PA group showing higher number of stretch attendances during the retention phase compared to those from PA group (3.30 ± 0.72 vs. 7.39 ± 1.37, *P* < 0.05). When comparing the freezing time (see Figure 6c), no changes were observed (72.10 ± 2.90% vs. 76.71 ± 2.87%, *P* > 0.05).

### 3.4 Freezing time in the retention phase of PA correlates with CB_1_ receptor activity in the NBM

**FIGURE 7:**
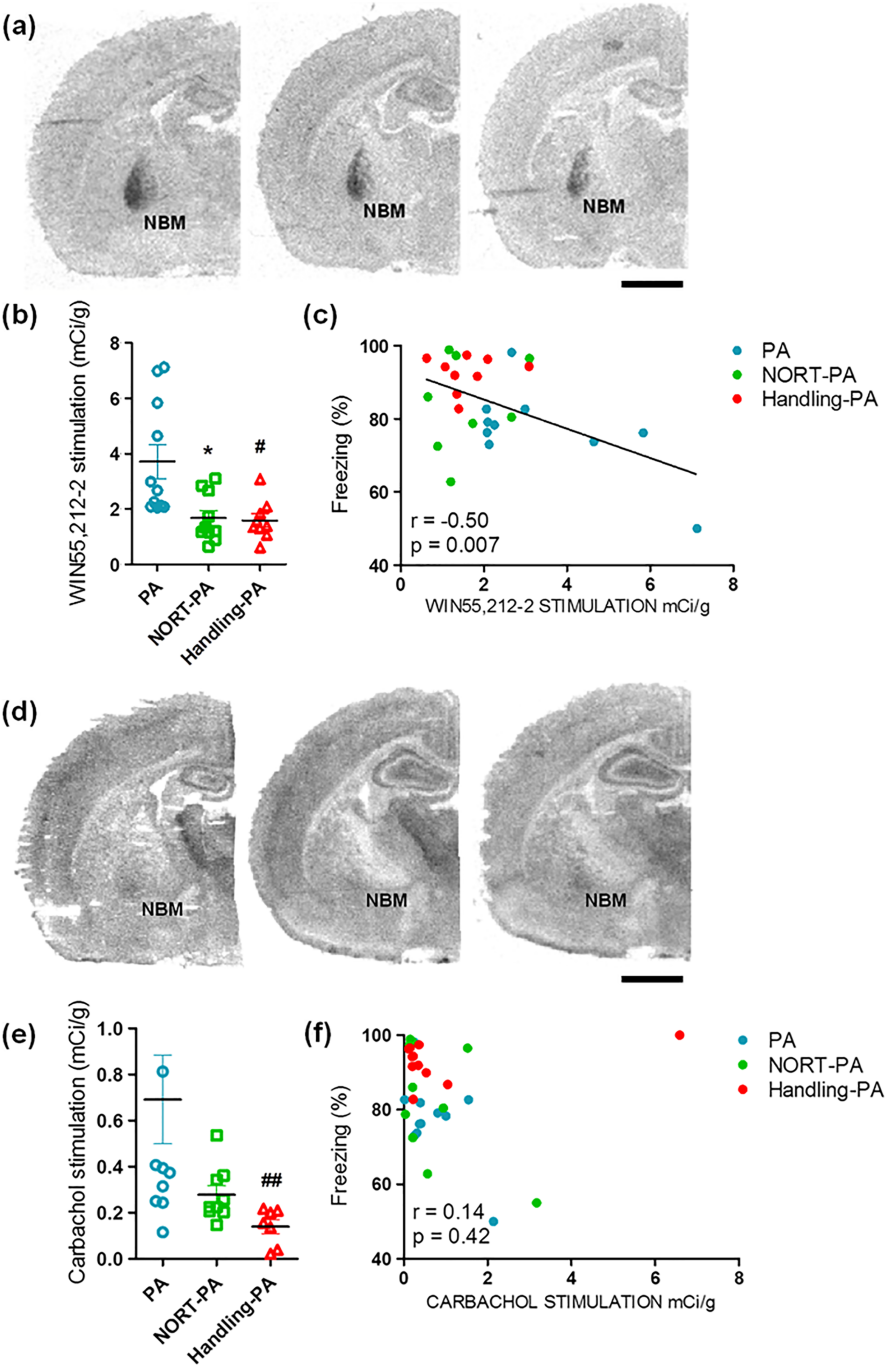
(a) Representative autoradiograms of PA, NORT‐PA, and Handling‐PA groups in rat coronal sections that show [^35^S]GTPγS stimulated by WIN55212‐2 in the NBM. Scale bar: 5 mm. (b) [^35^S]GTPγS binding stimulated by CB_1_ agonist WIN55212‐2 in the NBM. Significant differences were found between PA and NORT‐PA groups and PA and Handling‐PA groups (Kruskal–Wallis test, post‐hoc test Dunn's multiple comparison, H(2) = 11.13, *P < 0.05 PA vs. NORT‐PA, †*P* < 0.05 PA vs. handling‐PA). (c) Correlation between [^35^S]GTPγS binding stimulated by CB_1_ agonist WIN55212–2 in the NBM and the percentage of freezing time in the retention phase of PA (Spearman's test, *P* = 0.42, *r* = −0.50). (d) Representative autoradiograms of PA, NORT‐PA, and Handling‐PA groups in rat coronal sections that show [^35^S]GTPγS stimulated by carbachol in the NBM. Scale bar: 5 mm. (e) [^35^S]GTPγS binding stimulated by M_2_/M_4_ agonist carbachol in the NBM. Significant differences were found between PA and handling‐PA groups (Kruskal–Wallis test, post‐hoc test Dunn's multiple comparison, H(2) = 12.58, *P* > 0.05 PA vs. NORT‐PA, ††*P* < 0.01 PA vs. Handling‐PA). (f) Correlation between [^35^S]GTPγS binding stimulated by M_2_/M_4_ agonist carbachol in the NBM and the percentage of freezing time in the retention phase of PA (Spearman's test, *P < 0.05, *r* = 0.14)

The [^35^S]GTPγS binding stimulated by CB_1_ agonist WIN 55,212‐2 was measured in brain slices of naïve Sprague–Dawley male rats from PA, NORT‐PA and Handling‐PA groups. The aim was to observe the possible modulation of cannabinoid signaling induced by the different batteries of behavioral tests administered to the rats. The G protein‐coupled CB_1_ receptor activity induced by the CB_1_ agonist WIN 55,212‐2 was significantly higher (Kruskal–Wallis test; post‐hoc test Dunn's, *P < 0.05) in PA group than in NORT‐PA and Handling‐PA groups in the NBM (see Figure 7b). Moreover, the activity induced by the CB_1_ agonist WIN 55,212‐2 in the NBM correlated with the percentage of freezing time in the retention phase of PA (Spearman's test, *P < 0.05, *r* = −0.50, Figure 7c). No significant differences were found in G protein‐coupled CB_1_ receptor activity induced by the CB_1_ agonist WIN 55,212‐2 in other brain areas.

The [^35^S]GTPγS binding stimulated by cholinergic and dopaminergic agonists, carbachol and rotigotine respectively, was also measured. Significant differences were found in the G protein‐coupled M_2_/M_4_ receptors activity induced by carbachol in the NBM (see Figure 7e) between PA and Handling‐PA groups (Kruskal–Wallis test; post‐hoc test Dunn's, **P < 0.01). In this case, there was no correlation between the activity of M_2_/M_4_ receptors and the percentage of freezing time in the retention phase of PA (Spearman's test, *P* = 0.42, *r* = 0.14, Figure 7f). No significant differences were found in G protein‐coupled M_2_/M_4_ receptor activity induced by the M_2_/M_4_ agonist carbachol in other brain areas or in G protein‐coupled D_2_‐like receptor activity induced by the D_2_ agonist rotigotine.

## 4 DISCUSSION

Fear protects animals against perceived threats from the environment, triggering different reactions. Fight‐or‐flight response is often used when there is a possibility to avoid the threat (Mobbs & Kim, 2015). When that threat is seemingly inescapable, passive coping responses are adopted, for example, in the presence of a predator, some animals use a freezing strategy (Engel & Schmale, 1972).

In the present study, we evaluated the reaction of naïve Sprague–Dawley male rats to a learning and memory task under fear conditions, PA test. Firstly, the process of fear extinction was examined by exposing the rats to PA repeatedly throughout 7 months, in a non‐reinforced manner. Then, we studied the effect of animal handling and novelty‐seeking NORT on fear extinction (when performed after the aversive stimulus received in PA) and on fear expression (when performed before the aversive stimulus). Finally, we examined the correlates between the observed behavior and the activation of dopaminergic, cholinergic and endocannabinoid systems in key areas for the regulation of fear.

### 4.1 Fear extinction in PA is a robust and long‐term process

Our results provide evidence, for the first time, that the process of fear extinction in PA is a robust and long‐term process, lasting several months. When young adult rats were exposed to a mild foot shock and then exposed again to the same context every 2 months, in a non‐reinforced manner, a gradual decrease in retention latency was observed. Yet, even after 7 months, when the animals were close to middle‐age, approximately 40% of the rats still remembered the aversive stimulus and did not cross to the black compartment. To the best of our knowledge, it is the first time that fear extinction is measured for such a prolonged period of time in PA, in an experimental design mimicking stressor over a life‐span period from young to middle age. A previous study in mice showed a decrease in retention latency comparable to the one we have observed in just 45 days (El‐Ghundi et al., 2001). However, retention of PA response in those mice was first assessed 5 min after acquisition, whereas in our study, retention of passive avoidance response in rats was first assessed 24 h after acquisition. The temporal differences for the measurement of the mnemonist outcome in these paradigms may explain the subsequent differences observed in the duration of fear extinction, as immediate extinction after receiving the aversive stimulus attenuates spontaneous recovery in PA test (Briggs & Fava, 2016).

### 4.2 Handling and NORT accelerate fear extinction following an aversive stimulus

When we studied the effect of animal handling and NORT on fear extinction in rats that had received an aversive stimulus in PA, we observed that this process was fundamentally altered. When rats that had performed PA received a gentle handling protocol after it and were then exposed again to a non‐reinforced second retention of that test, they showed a reduced PA response, as measured by parameters such as retention latency, number of stretch attendances and freezing. These results are in line with previous studies that have indicated that handling has an anxiolytic effect in high‐arousal rodents. For instance, gentle handling of adolescent rats improved learning and memory and decreased anxiety, measured in the elevated plus‐maze test (Costa et al., 2012). Moreover, neonatal handling is beneficial for rodents in many aspects, such as reducing anxiety levels and bizarre behaviors, improving cognition and increasing playful responsiveness (Baeta‐Corral & Giménez‐Llort, 2014; Siviy, 2018). Neonatal handling decreases anxiety and stress response in rats in two‐way active avoidance test by significantly reducing the hippocampus and amygdala volumes (Río‐Álamos et al., 2017). Furthermore, neonatal handling can modulate the cognitive impairment and anxiety‐like behaviors in C57BL/6 mice with a severe perinatal hypoxic–ischemic brain injury, showing striking neuroprotective effects (Muntsant et al., 2019).

Additionally, we also report that the performance of NORT, in which rats also receive handling as part of the protocol, favors fear extinction. Rats which had performed NORT showed reduced retention latency and a smaller percentage of freezing time when they were exposed again to the context of PA in a non‐reinforced manner. To the best of our knowledge, this phenomenon has not been described before. However, the exposure of rats to environmental enrichment reduces anxiety in the open field test (Hines et al., 2012) and shares neurobiological mechanisms with neonatal handling (Fernández‐Teruel et al., 2002). Environmental enrichment activates structures of the medial prefrontal cortex, which is involved in the extinction of fear memories (Marek et al., 2018). We hypothesize that the interaction with different objects that is inherent to the performance of NORT could have an effect equivalent to environmental enrichment, thus resulting in increased fear extinction.

When we compare the results in experiments 1 and 2 we find that, interestingly, a process that, naturally, takes 7 months to happen, can be reduced to just over a week by performing NORT and, to a lesser extent, handling. Given that the performance of these procedures and the 7‐month time span deliver a similar result, we ruled out the possibility of performing these same procedures to the 7‐month‐old phenotype. In such case, we would be unable to properly evaluate the reduction of passive avoidance response caused by NORT or handling, because that response is already naturally reduced at that point.

### 4.3 Handling before an aversive stimulus exacerbates fear expression, whereas NORT reduces it

Then, we studied the effect of animal handling and the performance of NORT on fear expression following an aversive stimulus. In this case, as well, we found that the behavior of the rats fundamentally changed. When rats that had received a gentle handling were exposed to PA, they showed increased PA response, as measured by parameters such as retention latency, number of stretch attendances and freezing.

Moreover, these rats exhibited active escape behavior, by trying to jump out of the shuttle box several times. This kind of stress‐induced active avoidance response is not expected in PA, where animals learn to avoid an environment in which an aversive stimulus was previously delivered by staying still and not by actively moving away from that environment (Cimadevilla et al., 2000). In fact, freezing interferes with active escape learning in rats (Cain & LeDoux, 2007). Additionally, surges of autonomic arousal that are behind escape behavior are associated with fear, whereas avoidance behavior is associated with anxiety (Stahl, 2015). Thus, it can be concluded that the behavior observed in Handling‐PA group of rats is an exacerbated, fear‐motivated response to PA, indicative that handling done to low‐arousal rats before receiving an aversive stimulus acts as a stressor, increasing fear expression after the stimulus. To the best of our knowledge, the effect of gentle handling in naïve, low‐arousal rats, has never been studied before.

As opposed to handling, the performance of NORT before PA in naïve rats caused a significant reduction in passive avoidance response, considering parameters such as retention latency, number of stretch attendances and freezing. While handling is inherent to NORT protocol, it is possible that the interaction with different objects, which might be acting as some kind of environmental enrichment, is behind the observed behavior. As it has been previously mentioned, environmental enrichment has been shown to reduce anxiety (Hines et al., 2012), and behavioral despair (Torres‐Lista & Giménez‐Llort, 2015).

### 4.4 The freezing time in the retention phase of PA correlates with CB_1_ receptor activity in the NBM

Finally, in order to explain the mechanism by which NORT and handling were able to alter passive avoidance response, we then decided to analyze the neurochemical correlates of these behaviors. Although it could have been interesting to also study the neurochemistry of the original 7‐month old phenotype, these rats received PA once and nothing else for 7 months, other than non‐reinforced exposure to PA every 2 months. In the absence of NORT or handling, we would not expect any changes in these rats and, if there were some, it would be difficult to attribute the observed changes to the aversive stimulus received 7 months ago, rather than aging.

The activity of dopaminergic, cholinergic and cannabinoid receptors was studied, since there is evidence that interaction of these systems modulates fear expression. Previous studies have described that cannabinoid‐cholinergic interaction might be responsible for processing aversive memories and anxiety‐like behaviors (Fogaça et al., 2016; Manuel et al., 2016). Similarly, cannabinoids regulate dopamine activity in projections from several areas, including the mPFC, striatum and subthalamic nucleus, profoundly affecting the mesolimbic system, which regulate aversive memories (Ney et al., 2021).

In spite of the robust evidence indicating that dopaminergic signaling plays a role in fear response regulation, contributing to the expression and acquisition of fear memories (Guarraci et al., 1999), in this study we do not report changes in G protein‐coupled receptor‐mediated activity evoked by D_2_ agonist rotigotine, suggesting that the observed behavior might not be directly regulated by the dopaminergic reward system.

To the contrary, our results confirm the involvement of cannabinoid signaling in these behavioral modulations, as specific changes in the activity of CB_1_ receptors were found. In particular, PA group showed an increase in cannabinoid signaling in the NBM compared to NORT‐PA and Handling‐PA groups. Moreover, we found a negative correlation between CB_1_ receptor activity in the NBM and the percentage of freezing time. The experimental groups that were somehow handled before performing PA, Handling‐PA and NORT‐PA groups, showed a higher percentage of freezing time and a lower cannabinoid activity. Conversely, the group that was not handled before performing PA showed the lowest percentage of freezing time and the highest cannabinoid activity in the NBM.

These results are in line with recent studies that have demonstrated that the endocannabinoid system plays a key role in the regulation of anxiety and fear (Vickstrom et al., 2020; Wright et al., 2020). CB_1_ receptor‐mediated signaling is centrally involved in the behavioral adaptations that occur after the acquisition of aversive memories (Lutz, 2007), as it happens in PA. In line with that, CB_1_ receptor agonists WIN 55,212‐2 and Δ^9^‐tetrahydrocannabinol reduce anxiety‐like behavior in mice in elevated‐plus maze, further supporting the anxiolytic role of endogenous cannabinoid molecules (Patel & Hillard, 2006). In contrast, in mice which performed elevated‐plus maze and were treated with CB_1_ receptor antagonist rimonabant, this drug had an anxiolytic effect, suggesting that a high level of CB_1_ receptor activity increases, instead of reducing, anxiety‐like behavior (Griebel et al., 2005).

These seemingly contradictory effects of CB_1_‐mediated cannabinoid signaling may be explained by the fact that the effect of cannabinoids in the regulation of anxiety and fear depends on the dose, the duration of the treatment and the preexisting conditions of the subject (Maldonado et al., 2020). For instance, high doses or long‐term cannabinoid treatments increase anxiety levels both in humans and rodents, whereas an acute administration or low dose treatments with THC, among other cannabinoids agonists, relieve anxiety‐related behavior (Van Ameringen et al., 2020). Similarly, although a preexisting state of low arousal allows anxiety relief after potentiation of cannabinoid signaling in the amygdala, a state of high arousal prevents this effect (Morena et al., 2016). A study performed using the elevated plus maze test with rats in low and high arousal conditions demonstrated that pharmacologically‐induced elevations of endogenous cannabinoids decreased anxiety under low arousal conditions, but not with high arousal conditions (Morena et al., 2016). Similarly, we recently demonstrated that anxiety‐like responses in PA in 7‐month‐old 3xTg‐AD mice could be mediated by CB_1_ receptor hyperactivity in the basolateral amygdala. This behavior was restored to control levels after pharmacological desensitization of CB_1_ receptors in that area (Llorente‐Ovejero et al., 2018). These results suggest that the cannabinoid system plays a key role in the regulation of anxiety and fear expression, but this regulation is dependent on the basal state of arousal of the animals.

Additionally, we also report an increase in cholinergic signaling in PA group, compared to NORT‐PA and Handling‐PA groups, but no direct correlation was found between cholinergic activity in the NBM and the percentage of freezing time. This increased cholinergic signaling was observed in the same region where cannabinoid signaling also augmented, suggesting a crosstalk between both systems for the regulation of these processes, in line with previous results. CB_1_ receptors present at the presynaptic terminals of medial habenular (MHb) axons control the expression of aversive memories by selectively modulating cholinergic transmission (Soria‐Gómez et al., 2015). Furthermore, in the hippocampus, a region that has been described to be involved in the consolidation and maintenance of fear memories (Broadbent & Clark, 2013), CB_1_ receptors present on cholinergic neuronal projections have been described to control acetylcholine release (Degroot et al., 2006).

## 5 CONCLUSION

In conclusion, our results provide evidence that naturally occurring decrease in passive avoidance response after an aversive stimulus in naïve rats is a robust and long‐term process, lasting for up to 7 months. Whether this decrease in passive avoidance is a consequence of a learnt fear extinction process or is reflecting a decay of memory across time is difficult to state. Further studies, including a control group given a single PA retention phase at a long interval, are necessary to clarify this point. However, our results do show that this decrease in passive avoidance can be modulated and accelerated via different behavioral procedures, such as gentle handling of the animals and performance of NORT. In addition, we also report the effect of these procedures on fear expression following an aversive stimulus. Gentle handling provokes exacerbated fear‐motivated escape response when it is done before the aversive stimulus is delivered and rats are in a low arousal state, whereas NORT reduces fear expression following the same stimulus. We also identified the role of CB_1_ receptor‐mediated signaling and its possible interplay with increased cholinergic neurotransmission in the NBM in the modulation of passive avoidance response which can provide an experimental scenario to further study other neurobiological mechanisms involved in the development of fear response. Further studies analyzing the state of the aforementioned neuronal circuits in the groups where rats performed NORT or received handling after the aversive stimulus, as well as studying how these behaviors change using female rats, would provide new insights into the neurochemical regulation of passive avoidance response and its decay over time.

## CONFLICT OF INTERESTS

The authors declare that they have no known competing financial interests or personal relationships that could have appeared to influence the work reported in this paper.

## AUTHOR CONTRIBUTIONS

Iker Bengoetxea de Tena, Lydia Giménez‐Llort, Alberto Llorente‐Ovejero, Marta Moreno‐Rodríguez, Jonatan Martínez‐Gardeazabal, Sergio Monge‐Benito and Rafael Rodríguez‐Puertas contributed to the conception, design, and experiments of the study, performed the statistical analysis, and wrote the manuscript. All authors contributed to the manuscript revision, read, and approved the submitted version.

### PEER REVIEW

The peer review history for this article is available at https://publons.com/publon/10.1111/ejn.15642.

## Supporting information




**Data S1.** Supporting information

## Data Availability

The data that support the findings of this study are available from the corresponding author upon reasonable request.
